# Exploring how stay-at-home orders during the COVID-19 pandemic impedes engagement along the HIV/AIDS care continuum in public hospitals of Southwest Ethiopia: a qualitative study

**DOI:** 10.3389/fpubh.2024.1273448

**Published:** 2024-06-17

**Authors:** Abraham Tamirat Gizaw, Mengistu Abayneh

**Affiliations:** ^1^Department of Health, Behavior and Society, Faculty of Public Health, Institute of Health, Jimma University, Jimma, Ethiopia; ^2^School of Medical Laboratory Sciences, Mizan-Tepi University, Mizan Teferi, Ethiopia

**Keywords:** COVID-19, PLWHA, continuum of care, HIV/AIDS, adherence

## Abstract

**Introduction:**

COVID-19 has rapidly spread across the world. In March 2020, shortly after the first confirmed case of COVID-19 in Ethiopia in March 2020, the government of Ethiopia took several measures.

**Purpose:**

This study aims to explore how stay-at-home orders during the COVID-19 pandemic hinder engagement with HIV/AIDS care in public hospitals in Southwest Ethiopia. Additionally, we aim to explore the psychosocial challenges faced in accessing services during stay-at-home orders.

**Methods:**

A descriptive qualitative study was conducted from 20 May to 3 June 2020, using semi-structured, in-depth interviews. In total, 27 study participants were recruited from purposively selected people living with HIV/AIDS (PLWHA) who had experienced delays, declines, or discontinuation of care after COVID-19 was confirmed in Ethiopia on 13 March 2020. The participants were interviewed over the phone and their responses were audio-recorded. Data were transcribed verbatim, translated, and analyzed using inductive thematic analysis in the Atlas ti.7.1 software package.

**Results:**

The main themes and sub-themes that emerged were psychosocial issues (such as depression, hopelessness, and fear), risk perception (including high risk, susceptibility, and severity), forceful enforcement of stay-at-home orders (such as police beatings, community leaders disgracing, and influence of families and relatives), socioeconomic factors (such as stigma, religion, and transportation costs), misinformation about COVID-19 (such as lockdowns and ART stock-outs), and healthcare factors (such as inadequate health information and long distances to healthcare facilities).

**Conclusion:**

Overall, these findings were similar to the challenges experienced by PLWHA in adhering to the recommended continuum of care. However, there are additional factors due to COVID-19, such as misinformation and the forceful implementation of the stay-at-home-orders, that impede the continuum of care. Therefore, it is important to strengthen information, education, and communication.

## Introduction

People living with HIV/AIDS in Ethiopia face numerous challenges that impede their access to proper medical care and support. Stigma and discrimination against individuals with the virus are widespread in the country, causing many to hide their status due to fear of being socially rejected. This lack of openness about their condition can prevent them from seeking timely treatment, resulting in the progression of the disease. Furthermore, HIV/AIDS is often associated with risky behaviors such as drug use and promiscuity in Ethiopian society, leading to blame and judgment from their communities for those affected by the virus. This further contributes to the isolation and marginalization of individuals living with HIV/AIDS, making it difficult for them to obtain the necessary resources for their health and wellbeing (1–3).

The high levels of poverty in Ethiopia pose significant challenges for individuals living with HIV/AIDS. Many individuals affected by the virus struggle to afford basic necessities such as food, housing, and transportation to medical appointments. Without access to adequate nutrition and a stable living environment, their ability to adhere to a strict medication regimen and maintain overall health is compromised. Moreover, the lack of affordable healthcare services in rural areas of the country exacerbates the challenges faced by those living with HIV/AIDS. These factors highlight the urgent need for increased awareness, education, and support for individuals living with HIV/AIDS in Ethiopia. It is crucial to ensure that they can access the care and resources necessary to effectively manage their condition (4–6).

The COVID-19 pandemic has not only affected physical health but has also resulted in adverse effects on the psychological wellbeing of people worldwide (7). As healthcare systems become overwhelmed and people avoid visiting health facilities due to restrictions and fear of exposure, there is a rise in preventable disease-related deaths, both directly and indirectly. This pandemic has significantly affected healthcare systems worldwide, especially in low- and middle-income countries that rely heavily on pharmaceutical imports to meet the needs of individuals with chronic diseases (8).

Studies have shown that African countries, especially Egypt, Algeria, South Africa, Ethiopia, and Nigeria, are facing a high risk of importing COVID-19. Ethiopia, in particular, has reported widespread disruptions in international travel and a shortage of medical supplies, making it difficult to provide adequate medical care. This has posed significant challenges for people living with HIV/AIDS (PLWHA) who are unable to access healthcare facilities for their routine care and medication management during the current COVID-19 pandemic (9, 10).

Although there is no strong evidence suggesting that PLHIV are at a higher risk of severe COVID-19 if they acquire it, people with underlying conditions may be more vulnerable to the infection (11). The COVID-19 pandemic has also disrupted HIV care services, including the loss of or limited access to healthcare services and HIV funding. Sub-Saharan Africa is, in particular, susceptible to COVID-19 due to its weak healthcare infrastructure, low clinician-population ratios, limited laboratory capacity, and underlying conditions, including malnutrition, anemia, and chronic respiratory conditions (12–17). Ensuring medication adherence during COVID-19 is essential in reducing the burden among immunosuppressed individuals, particularly PLWHA. The World Health Organization recommends that PLWHA should start ART immediately. PLWHA without COVID-19 symptoms should be initiated on ART on the day of diagnosis, preferably on a DTG regimen, and provided with a 3-month supply at initiation to reduce the need to visit healthcare facilities during COVID-19 (18–21). This study recognizes that people living with HIV/AIDS face a dual challenge during the COVID-19 pandemic and government-imposed stay-at-home orders. Globalization and the recurring emergence of infectious diseases highlight the need to develop strategies to support populations with comorbidities. Therefore, this study seeks to understand the challenges of engagement in HIV/AIDS care during the COVID-19 pandemic in Southwest Ethiopia.

## Methods

### Study setting and period

This study was conducted in public hospitals in Southwest Ethiopia. Specifically, this study was conducted in four selected public health hospitals from May 20 to June 3, 2020 in Jimma University Medical Center, Bonga Hospital, Mizan-Tepi University Teaching Hospital, and Tepi Hospital.

### Study approach

A descriptive qualitative study was conducted to examine the challenges that hinder the HIV/AIDS continuum of care. The qualitative descriptive method is a research approach that aims to represent the perspectives of a specific population under study. This approach allows researchers to focus on the words and meanings provided by participants and to present a thorough overview of a phenomenon in easy-to-understand language. Naturalistic inquiry involves studying something in its natural environment, without predetermined variables or theoretical biases. In qualitative descriptive studies, researchers analyze data at the surface level of words and events, rather than delving into the deeper levels explored in grounded theoretical, phenomenological, ethnographic, or narrative studies.

### Recruitment

A purposive sampling technique was used to select participants for IDIs. A total of 27 in-depth interviews were conducted through phone calls and the responses were recorded with permission to comply with the social distancing protocol. The participants were included based on the following criteria: those who had delayed, declined, or discontinued antiretroviral therapy after COVID-19 was confirmed in Ethiopia on 13 March 2020, aged ≥ 18 years, and who started ART medication before 6 months and above. Selection, data collection, and analysis were continued until saturation. Table 1 summarizes the socio-demography of the IDI participants.

**Table 1:** Socio-demographic characteristics of IDI participants who take part in phone calls for in-depth interviews, 2020.

**Characteristic**	**Category**	**Number**
Public hospital involved	Jimma University Medical Center	7
	Bonga Hospital	7
	Mizan-Tepi University Teaching Hospital	7
	Tepi Hospital	6
Sex	Male	17
	Female	10
Age	21–30	3
	31–40	6
	41–50	12
	51–64	4
	65 and above	2
Marital status	Single	7
	Married	12
	Divorced	3
	Widowed	5
Educational status	Primary	9
	Secondary School	7
	Senior Secondary School	6
	College Certificate/Diploma	2
	University degree	3
Month on ART regimen	6 months	2
	7–12 months	4
	13–18 months	3
	19–24 months	12
	Above 24 months	6
Types of non-adherence	Delayed	6
	Declined or discontinued	21
Duration of non-adherence	One month	3
	Two month	13
	Three month	9
	Four month	2
Occupation	Housewife	8
	Farmers	12
	Merchants	5
	Government employee	2

### Data collection procedures

Data were collected using a semi-structured guide (10 open-ended questions with probing lists) developed from different studies in the English language and then translated into Afan Oromo and Amharic languages and back-translated into English by independent translators for consistency in meaning. The guide was prepared to address the research question starting with general and moving to specific, taking into consideration the local knowledge and cultural sensitivities. The guide was developed to cover topics related to challenges to the HIV/AIDS continuum of care during the stay-at-home order of the COVID-19 pandemic.

### Data analysis

An inductive thematic analysis was used to analyze the data. After listening to the audio-recorded material, verbatim transcription was performed by the researcher simultaneously to ensure data saturation (16). The transcriptions were then checked for completeness and consistency. Then, the transcripts of IDI conducted among people living with HIV/AIDS were translated from Afan Oromo and Amharic to English by the researchers. The translations were read and re-read to extract important statements from the description, and then the coding was made by the researchers. To develop the codebook, line-by-line coding was conducted separately by the principal investigator on the ATLAS.Ti.7.1 software package (https://atlasti.com/). Potential categories and themes were developed by clustering subcategories and categories, respectively, which answers the research questions. The researchers repeated the coding system after the first codebook was developed while refining the codebook, categories, and themes. Finally, results were presented with seven major themes, sub-themes, and quotations derived from the data.

### Rigor

In order to ensure trustworthy qualitative results, it is important to employ experienced data collectors. Detailed and descriptive explanations, along with the use of a codebook, were implemented to ensure transferability for comparative purposes. Dependability was ensured by outlining objectives and having an expert review every stage. Detailed data collection and prevention of researcher bias resulted in a high level of neutrality. An audit trail was created to document each step of data analysis. The lead researcher in this study was a PhD holder and assistant professor of public health who teaches qualitative research to postgraduate students. The other authors are medical doctors working in ART clinics of Jimma University Medical Center (JUMC), Assistant professor of microbiology from Mizan-Tepi University, PhD in health communication from Dilla University, and Master of public health working at the International Institute for Primary Health Care with many years of research experiences in qualitative research.

### Ethical considerations

Ethical approval was obtained from the Jimma University Research Ethical Review Board, Ethiopia. The rights of research participants were maintained by ensuring non-maleficence and underscoring the benefits of the study. Study participants were informed adequately about the purpose of the study, participated voluntarily, and had the right to participate or withdraw at any time. In order to ensure their privacy and autonomy, codes were given to participants and were informed that the study uses the codes in place of their names in connection to the study findings or in their answers in interviews. Time was given to them to reflect and provide a detailed explanation of the issue. Due to the home-at-stay-order of the Ethiopian government, verbal consent was obtained from all the participants. No participant has refused to participate in the study and to be recorded. All participants have completed the entire sessions of the IDIs.

## Results

### Stay-at-home order and its impediment to the continuum of HIV/AIDS care during the COVID-19 pandemic

The findings of this study were organized under seven main themes and sub-themes. The main themes that emerged from the analysis of IDIs with PLWHA were psychological reactions, risk perception, forceful stay-at-home-order enforcement, economic factors, social factors, misinformation, and health services (Table 2).

**Table 2:** Themes and sub-themes.

**Themes**	**Sub-themes**
Psychological reactions	Depression
	Hopelessness
	Fear
Risk perception	Susceptibility to COVID-19
	Severity on PLWHA
Forceful stay-home-order enforcement	Police beating
	Community leader disgracing Families and relatives influence
Economic factors	Transportation cost
	Employment status
Social factors	Stigma
	Religion
Misinformation	Lockdown
	ART medication stock out
Health services	Inadequate health information
	Distance from the hospital
	Focus shifted to COVID-19

### Theme 1: psychosocial reactions

Participants reported experiencing various psychosocial reactions due to COVID-19, such as depression, hopelessness, and fear. In March 2020, when the first testing and confirmation of COVID-19 testing occurred in Ethiopia, the media started reporting on the COVID-19 pandemic at local and national levels. The government then imposed a stay-at-home order and used personal protective equipment. These issues are alarming for individuals with co-morbidities, such as being HIV/AIDS positive. Depression, hopelessness, and fear were the major issues that were faced by individuals due to the COVID-19 pandemic. The majority of individuals who took part in the IDI expressed experiencing various forms of depression, hopelessness, and fear related to COVID-19.

“*…When I heard COVID-19 entered Ethiopia, I feel like it is the end of the world because I started to panic after being infected with the HIV/AIDS virus. I thought it would infect me; I did not want to go for medication to the hospital and refill my medication. I discontinued my ART medication…” (IDI-3)*

“*… depressed and became hopeless that emergence of COVID-19 as the world threat and hearing information that people living with HIV/AIDS are more vulnerable to the infection [COVID-19] …” (IDI-5)*

“*…Totally depressed and became hopeless that emergence of COVID-19 as the world threat and hearing information that people living with HIV/AIDS are more vulnerable to the infection [COVID-19] …” (IDI-12)*

### Theme 2: risk perception

Most of the respondents perceived that they had a high risk of acquiring COVID-19 and the susceptibility and severity of COVID-19 in individuals living with HIV/AIDS due to comorbidity and immunity. The perception affected individuals to go out and refill their ART medication even though they had no stock left. Participants were concerned about the risks related to COVID-19.

“*…COVID-19 is dangerous because we have HIV/AIDS in our blood, we are less likely to survive it, and COVID-19 imposes great consequences on us…” (IDI-19)*

“*…Mostly the health professional tells us that we are at risk of getting COVID-19 when we are going for follow up. I constantly think of my immunity and when I hear about the seriousness of this emerging pandemic [COVID-19], I totally perceived that it will take my life…” (IDI-9)*

### Theme 3: forceful stay-at-home-order enforcement

In the middle of -March, the government of Ethiopia declared an emergency order due to COVID-19, to contain the spread of COVID-19, which is to stay at home. Initially, due to the disobedience of people, the police and other responsible individuals were obliged to enforce the order. However, there was a problem with identifying who should be allowed to go out and who should not. Some individuals were subjected to beating and disgrace. Therefore, due to the fear of police beating, community leader disgracing, and families and relative influence to not go out for ART medication refills, led to discontinuation of the ART. The participants expressed forceful law enforcement that hindered them not to refill their medications.

“*…I know my medication is finished but if I go to ART clinic, the police can beat me out of the house and refrain from going. Also, the local community leader disgraces me. The option is discontinuing my ART medication for the time being…” (IDI-27)*

### Theme 4: economic barriers

The respondents expressed their opinions related to transportation costs and employment status. Participants complained that they were unable to afford the cost due to the double payment to keep physical distancing during bus travel to the ART center. Most of the participants were unemployed and were daily breadwinners. Due to stay-at-home-order, they lost their income and were even unable to eat once a day. The participants emphasized the economic barriers as the major problem that affected their medication adherence.

“*…I don't even have to eat, let alone take my two-way ART medication to pay for my transportation. I was builder and due to COVID-19 lockdown, I have lost my jobs and due to transportation cost, I discontinued ART medication…. “(IDI-6)*

“*…As it is known that COVID-19 has economic impact, I have also lost my job due to the disease [COVID-19]. Therefore, it is difficult to get money for transport payment, and I have missed my appointment for medication refill and for other follow-ups…” (IDI-7)*

### Theme 5: social factors

The other participants responded that social factors, such as stigma and religion, were considered challenges to the HIV/AIDS continuum of care. The major challenge was the stigma experienced by PLWHA. For instance, they easily identify when they went to the service because others have still stayed in their homes. Religion is another contributing factor to discontinuing the HIV/AIDS continuum of care. Examining the churches within the community and drinking holy water locally called “tsebel” by Orthodox Christianity followers and “Dua” group prayer among Muslim religion followers as an alternate option to the continuum of care. The participants mentioned in their expression that religion and stigma are the major barriers to adhering to the continuum of care.

“*…I am currently out of antiretroviral therapy medication, but if I go to the hospital, my neighbor may suspect that I have some special disease that enforced me to go for treatment during this critical time. Once she knew my status that I am HIV/AIDS positive, she could stigmatize me. Due to this, I discontinued the ART medication and started drinking “tsebel” from our neighborhood church…” (IDI-24)*

“*…currently the world is without solution, no medication that cures HIV/AIDS and even for COVID-19. The only solution is in the hands of God. Therefore, it is better to go to holy water for getting a solution…” (IDI-8)*

### Theme 6: misinformation

Other IDI participants explained that there were various forms of rumors related to antiretroviral therapy medication supply issues. The participants were explained that due to the lockdown, there is no ART supply and the medication is stocked out. Because of the misinformation, most PLWHA failed to refill their medication on their appointment date. The participants stated that the misinformation obtained from different sources affected their follow-up.

“*…I heard that there is no ART medication and health professionals are all working on COVID-19 prevention and treatment. Due to this I missed my appointment to go to ART clinics…” (IDI-21)*

### Theme 7: health service factors

The IDI participants mentioned that there are some challenges including inadequate information that the PLWHA regarding the importance of medication adherence, especially factors such as inadequate health information, distance from hospitals, and focus shift to COVID-19.

“*…due to fear of stigma and discrimination, I have ART follow-up in hospital out of my district [zone]. I feel the distance when I lack money to pay for transportation. Therefore, I decided to decline my appointment…” (IDI-26)*

## Discussion

This study attempted to explore how stay-at-home orders during the COVID-19 pandemic impede engagement along the HIV/AIDS care continuum and identified psychosocial factors, such as depression, hopelessness, and fear, as the sub-themes from the in-depth interviews of the participants. The findings of this study are in line with a study conducted on factors influencing medication adherence, which was found to be in adherence with greater stress, depression, and symptom distress (15). This study was also in line with a similar study conducted in the United States during the COVID-19 pandemic that aged individuals and those with HIV have also endured significant trauma due to AIDS, contributing to high rates of depression (17).

During the COVID-19 pandemic, those with co-morbidities, such as HIV/AIDS, have a high risk of being infected with COVID-19 due to their lower immunity (18). Participants in this study reported that they had high-risk perceptions. They perceived that they had a high risk of being infected with COVID-19 and severity due to HIV/AIDS. This study has similar findings to a study on the syndemic burden of COVID-19 in people living with HIV, which showed that the emergence of COVID-19 creates another health burden for people living with HIV/AIDS who face multiple morbidities and may be at heightened risk for severe physical health illness from COVID-19 (19). Another study revealed that staying home during the COVID-19 pandemic prevented seeking services. The long-term impacts of this and related measures must also be considered in terms of their effects on the health of PLWHA (20, 21).

Forceful stay-at-home-order enforcement was the challenge that influenced the HIV/AIDS continuum of care. Police and community leaders misuse their power by beating and disgracing (22). They lost some high-risk groups to go and use services like PLWHA. The participants in this study expressed that they discontinued their HIV/AIDS continuum of care due to the beating by the police officials, the disgracing of the community leaders, and family and relative pressure not to go out of the home. Similar evidence has been reported in several Indian states, and photographs and videos show police officials beating people who are trying to get essential supplies (23, 24).

The study found that economic barriers were the other theme that had a significant impact on the continuum of care for PLWHA. The subthemes related to economic factors, such as transportation cost and employment status, were other barriers to PLWHA. During the COVID-19 pandemic, people are obliged to pay double for transportation to keep a physical distance. Those who were even unable to pay for themselves will have no option other than discontinuing the continuum of care for HIV/AIDS. These findings of this study were aligned with a study performed in Zambia, which identified economic factors, such as transportation and employment status (22), as barriers to ART adherence.

In this study, the participants mentioned social factors in the in-depth interviews. HIV/AIDS-related stigma and religion were barriers to seeking a continuum of care among people living with HIV/AIDS. This study had similar findings to the study done in Zambia, which considered religious beliefs and HIV-related stigma and discrimination as barriers to medication adherence (22). However, the study conducted in the Democratic Republic of Congo showed religious beliefs as both a barrier and facilitator of ART adherence (21).

Misinformation is another theme identified from the in-depth interviews of the participants. The subthemes identified were lockdown and going stock out of ART medication. The rumor spread contributed to the discontinuation of ART drugs. This study was supported by another study in which the participants expressed fear of how the COVID-19 pandemic will disrupt HIV care services, such as the loss of or restricted access to healthcare services (23). The supply-related fear was because low-income countries depended on the supplier, and fear is anticipated because the COVID-19 pandemic absorbed the attention of the developed countries. This is evidenced by other studies that stated LMICs often rely heavily on pharmaceutical imports, and the impact of the pandemic may be felt when essential medicines are unavailable and inaccessible to meet the needs of all, especially those with chronic diseases (9).

Health service factors are another major theme identified from the IDI of the participants, including inadequate information, distance from hospitals, and focus shifted to COVID-19. The response of PLWHA showed that they have limited information related to the continuum of care given to HIV/AIDS and its importance in combatting the comorbidity related to COVID-19. The focus shift to COVID-19 allowed the healthcare provider to emphasize less on chronic diseases, such as HIV/AIDS. Low cue to action from the provider side allowed individuals to neglect the continuum of care provided to HIV/AIDS. The finding is similar to a study performed on determinants of adherence in Africa that reported that challenges to ART medications were health care systems (24). In a study that conducted differentiated service delivery during COVID-19, a 3-month supply was provided at initiation to reduce the need to visit a health facility during COVID-19, with greater emphasis placed on initiation outside of facilities, ensuring the continuum of care for HIV/AIDS during the COVID-19 pandemic (25, 26).

The strength of this study lies in the fact that it provides an in-depth understanding of the barriers to ART adherence during the pandemic. Second, as this is a qualitative study, it goes beyond the research question and provides practical evidence for the teaching of qualitative study as a characteristic of flexibility.

## Conclusion

This study found that psychosocial factors, risk perceptions, forceful stay-at-home order enforcement, economic and social factors, misinformation, and health care factors influenced ART medication adherence and other health-seeking behaviors of PLWHA. To ensure the continuity of HIV/AIDS care, it is crucial for the health system to focus on providing targeted health information and communication, timely appointment reminders through phone calls, and establishing a confidential home delivery system for ART medication until the end of the COVID-19 pandemic. Additionally, structured communication should emphasize the risks of not receiving treatment and encourage PLWHA to continue hospital follow-ups to mitigate the risk of contracting COVID-19. In shaping the future, it is important that guidelines should prioritize the needs of vulnerable segments of the population (PLWHA) before implementing laws that may isolate them from essential services during a public health crisis.

## Data availability statement

The original contributions presented in the study are included in the article/supplementary material, further inquiries can be directed to the corresponding author.

## Ethics statement

The studies involving humans were approved by Jimma University's Institute of Health Research and Postgraduate Office, Institutional Review Board. The studies were conducted in accordance with the local legislation and institutional requirements. Written informed consent for participation was not required from the participants or the participants' legal guardians/next of kin because The data was collected through phone calls during the panic time of the pandemic when stay-home order was enforced and physical contact was prohibited. Instead, verbal consent was obtained from all the participants.

## Author contributions

AG: Conceptualization, Data curation, Formal analysis, Investigation, Methodology, Software, Validation, Writing—original draft. MA: Conceptualization, Data curation, Investigation, Supervision, Validation, Visualization, Writing—review & editing.
